# Self‐Healing Ability of Perovskites Observed via Photoluminescence Response on Nanoscale Local Forces and Mechanical Damage

**DOI:** 10.1002/advs.202204393

**Published:** 2022-12-01

**Authors:** Marco H. J. J. Galle, Jun Li, Pavel A. Frantsuzov, Thomas Basché, Ivan G. Scheblykin

**Affiliations:** ^1^ Chemical Physics and NanoLund Lund University Box 124 Lund 22100 Sweden; ^2^ Department of Chemistry Johannes Gutenberg‐University Duesbergweg 10‐14 55128 Mainz Germany; ^3^ Voevodsky Institute of Chemical Kinetics and Combustion Siberian Branch of the Russian Academy of Science Institutskaya 3 Novosibirsk 630090 Russia

**Keywords:** defects, metastability, photoluminescence, self‐healing, strain

## Abstract

The photoluminescence (PL) of metal halide perovskites can recover after light or current‐induced degradation. This self‐healing ability is tested by acting mechanically on MAPbI_3_ polycrystalline microcrystals by an atomic force microscope tip (applying force, scratching, and cutting) while monitoring the PL. Although strain and crystal damage induce strong PL quenching, the initial balance between radiative and nonradiative processes in the microcrystals is restored within a few minutes. The stepwise quenching–recovery cycles induced by the mechanical action is interpreted as a modulation of the PL blinking behavior. This study proposes that the dynamic equilibrium between active and inactive states of the metastable nonradiative recombination centers causing blinking is perturbed by strain. Reversible stochastic transformation of several nonradiative centers per microcrystal under application/release of the local stress can lead to the observed PL quenching and recovery. Fitting the experimental PL trajectories by a phenomenological model based on viscoelasticity provides a characteristic time of strain relaxation in MAPbI_3_ on the order of 10–100 s. The key role of metastable defect states in nonradiative losses and in the self‐healing properties of perovskites is suggested.

## Graphical abstract

Defects created in perovskite crystals by a local mechanical force are temporary. After several minutes they disappear leading to recovery of the initially quenched photoluminescence. The process is explained by treating the perovskite as a viscoelastic solid and relating nonradiative charge recombination to strain. The authors argue that the strain affects photoluminescence via stabilizing metastable super traps also thought to be responsible for photoluminescence blinking.

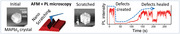

## 1 Introduction

Metal halide perovskites (MHPs) are semiconductors prepared by room‐temperature crystallization from precursor solutions.^[^ ^1^, ^2^, ^3^ ^]^ The power conversion efficiency of small area solar cells based on these materials has already reached 25%.^[^ ^4^ ^]^ One of the main features making perovskites so successful is the tolerance of their optoelectronic properties to chemical and structural defects inevitably formed during fast crystallization in solution and by kinetic competition between several intermediates.^[^ ^2^ ^]^ In addition to defect tolerance, MHPs were found to possess some features which can be loosely formulated as self‐healing properties.^[^ ^2^, ^5^ ^]^ It means that a partly degraded device (e.g., a solar cell) can restore its performance after several hours at “rest.” The most common demonstrations of self‐healing processes are recovery of photoluminescence (PL) from bleaching,^[^ ^5^, ^6^, ^7^, ^8^, ^9^, ^10^ ^]^ light‐induced PL enhancement,^[^ ^11^, ^12^ ^]^ and improvement of electronic properties of thin films after prolonged storage.^[^ ^13^, ^14^ ^]^ The combination of defect tolerance and the ability to heal spontaneously or under an external influence (e.g., light, current, etc.) are the crucial features opening the door for perovskites to develop into long lasting or “eternal” devices.^[^ ^15^, ^16^ ^]^

The feature of MHPs making self‐healing possible is the soft and dynamic ionic crystal lattice. It allows for ion migration and dynamic formation and annihilation of defect states. It is also very important that the formation energy for many defect states is higher than the energy needed to simply rebuilt the perovskite unit cell and avoid defect formation.^[^ ^2^, ^15^, ^17^, ^18^ ^]^ Self‐healing not only concerns defects and structures at the nanoscale, but also the repair of macroscopic cracks and even reconstruction of broken single crystals. The repair of broken mm‐sized single crystals requires the crystal fragments to be kept together for 24 h under pressures of around 1 MPa,^[^ ^19^ ^]^ while only a minute is needed to heal microcracks when the parts of a cracked film are pressed against each other.^[^ ^20^ ^]^ All these properties make MHPs, on the one hand, not very stable, on the other hand, they allow the materials and devices to recover their performance after degradation.

Although these effects are very prominent in MHPs, these materials are not unique in this context. For example, transient phenomena as reversible PL bleaching/enhancement under light soaking have been observed quite some time ago, for example for films of CdSe and CIGS materials and also have been related to defect metastability.^[^ ^21^, ^22^ ^]^ Recently, photoassisted PL recovery after bleaching was also observed in monolayer transition metal dichalcogenide (1L‐WS_2_) materials.^[^ ^23^ ^]^

MHP micro‐ and nanocrystals exhibit pronounced PL blinking and flickering.^[^ ^24^, ^25^, ^26^, ^27^, ^28^, ^29^, ^30^, ^31^ ^]^ When comparing classical semiconductor quantum dots^[^ ^21^, ^32^ ^]^ with MHPs we note that PL blinking and flickering is not only observed in MHP nanocrystals,^[^ ^33^ ^]^ but also in sub‐micrometer crystals, grains in thin films and even in micrometer‐sized objects.^[^ ^26^, ^30^, ^34^, ^35^ ^]^ The current understanding is that the PL intensity fluctuations occur due to the presence of very efficient, yet metastable nonradiative (NR) recombination centers, also called supertraps (Figure S1, Supporting Information). Due to the long‐range charge carrier diffusion in MHPs and large capturing and recombination cross sections of the supertraps, just one active supertrap can dominate the NR recombination in a whole sub‐micrometer MHP crystal.^[^ ^26^, ^36^ ^]^ Note that this mechanism, based on the efficient diffusion of the excited states toward the metastable quencher, is similar to the one frequently used to explain fluorescence blinking of single multichromophporic systems like *π*‐conjugated polymers and molecular aggregates.^[^ ^37^ ^]^ In general, one can also see PL blinking as random degradation/healing pathways which suggests that defect metastability and self‐healing are consequences of the same fundamental processes.

Recently, a lot of attention has been paid to crystal strain as a factor influencing the electronic properties of MHPs and the performance of MHP based devices.^[^ ^38^, ^39^, ^40^, ^41^, ^42^, ^43^, ^44^, ^45^, ^46^, ^47^, ^48^ ^]^ In addition, peculiarities of the crystallization kinetics and the conditions of the crystal growth can modulate crystal strain and thus electronic properties of the perovskite materials.^[^ ^49^, ^50^ ^]^ It was also suggested that lattice strain can enhance NR recombination rates by providing a driving force for defect formation.^[^ ^47^, ^51^, ^52^ ^]^ Moreover, the application of uniform pressure to MHP crystals showed that external pressure can decrease PL intensity, change PL decay kinetics, induce spectral shifts and eventually cause phase transitions in MHPs.^[^ ^41^, ^48^, ^53^, ^54^ ^]^

Considering the crucial importance of crystal strain as well as surface and bulk defects for charge carrier dynamics, atomic force microscopy (AFM) appears as a valuable tool to influence electronic properties of soft MHP materials by mechanical nanomanipulation. Indeed, the tip of an AFM cantilever can be used not only to probe the sample topography, but also to apply mechanical stress, to pull, stretch, and break organic molecules,^[^ ^55^, ^56^ ^]^ and even to mechanically modify surfaces using the tip as a “nanoknife.”^[^ ^57^, ^58^ ^]^ It is well‐known that the PL provides very rich information about charge recombination and defect states in MHPs.^[^ ^26^, ^27^, ^59^, ^60^, ^61^, ^62^ ^]^ Therefore, to monitor the effects of local nanoscopic mechanical manipulations on the PL, a promising experimental approach is correlative atomic force and confocal fluorescence microscopy.^[^ ^63^, ^64^, ^65^ ^]^ This technique has been successfully used to measure force‐induced spectral shifts of single organic dye molecules and inorganic semiconductor quantum dots.^[^ ^66^, ^67^ ^]^ Thus, the correlative microscopy provides a unique opportunity to create/influence defects by a mechanical force and probe their evolution by PL.

In this work, we combined AFM and confocal PL microscopy to study CH_3_NH_3_PbI_3_ (MAPbI_3_, MA = CH_3_NH_3_) polycrystalline perovskite microcrystals. The AFM tip was used not only to apply a localized anisotropic force, but also to scratch and even cut MAPbI_3_ crystals at the nanoscale. Despite such vigorous mechanical disturbances, we did not observe permanent quenching of the PL from the crystals. Initial complete PL quenching caused by the mechanical manipulation, was found to always disappear after several minutes leading to full recovery of the initial PL intensity. We show a clear connection between the tip‐induced quenching and PL blinking behavior, discuss possible physical mechanisms of self‐healing based on metastable NR‐ recombination centers and propose a phenomenological model of the effect based on viscoelasticity.

## 2 Experimental Section

### Sample Preparation

A 0.8 m MAPbI_3_ precursor solution was prepared by mixing 461 mg PbI_2_ (99% from Sigma‐Aldrich) and 159 mg MAI (99% from Sigma‐Aldrich) in 1.25 mL *γ*‐butyrolactone (GBL, ≥99% from Sigma‐Aldrich). The solution was stirred for 2 h at 60 °C. To prepare MAPbI_3_ microcrystals, the MAPbI_3_‐GBL precursor solution was diluted 1:2000 (concentration of 4 × 10^−4^ m). 20 µL of this solution were drop‐cast on a glass substrate. Finally, the sample was annealed at 80 °C for 20 min by placing the glass substrate on a temperature‐controlled heating plate. All preparations were carried out in ambient air. This method usually leads to polycrystalline perovskite particles of various size from tens of nanometers to tens of micrometers dispersed on the glass surface.

### Experimental Setup

**Figure 1:**
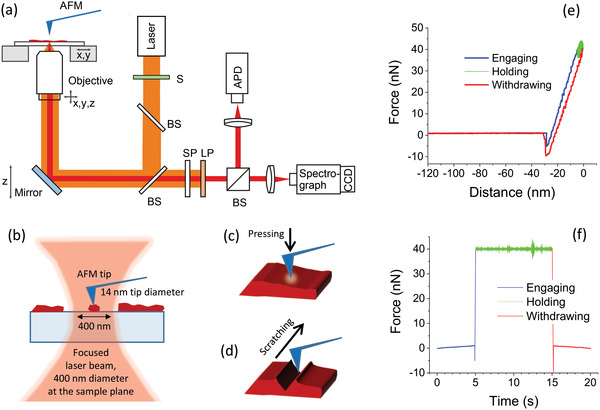
(a) Simplified sketch of the experimental setup consisting of a home‐built confocal PL microscope and a commercial atomic force microscope (AFM). S: shutter, BS: beam splitter, SP: short‐pass filter, LP: long‐pass filter, APD: avalanche photodiode, CCD: charged coupled device. (b) Relation between the excitation spot size (lateral ≈400 nm), the size of the perovskite crystals (from tens of nm to tens of micrometers) and the tip diameter (≈14 nm). (c) Application of force with the AFM tip. (d) Scratching of the sample with the AFM tip. (e) Force–distance curve which can be transformed into a time‐dependent force shown in (f).

The experimental setup (**Figure** **1**a) consisted of a home‐built inverted confocal microscope and a commercial AFM (mfp‐3d, Asylum Research) positioned above the microscope objective. Silicon tips with radii of about 7 nm (OPUS 240‐AC‐NA‐100, MikroMash) were used for probing topology and for applying a local force on the sample. The sample was excited by a CW diode laser (640 nm), resulting in 0.45 W cm^−2^ excitation power density at the sample plane after focusing the light by an oil immersion objective (1.4 NA, 100×, Zeiss). The objective could be moved by a three‐axis piezoelectric translation stage. The sample holder was placed on top of a two‐axis piezoelectric translation stage belonging to the AFM. The sample holder and the AFM head were designed to create an almost closed volume around the sample. A small hole in the sample holder allowed to generate a small constant flow of argon gas to displace the ambient air. Light emitted by the sample was collected by the same objective and sent through a short‐pass filter to block the NIR laser light used in the AFM and a long‐pass filter to remove the excitation light. Then the emission was guided to a photon‐counting detector (APD, SPCM‐AQRH‐15, Perkin Elmer) and a spectrograph equipped with a CCD camera. Except the lasers and the spectrograph with the CCD, the whole setup was placed on a vibration isolation table and temperature stabilized to 28 °C by a closed isolation box. The relative humidity of the air inside the box was around 30%; see more detailed description of the setup elsewhere.^[^ ^66^, ^67^ ^]^ It is noted that the AFM tip was probing/manipulating the top surface of the microcrystals, while the PL was excited and collected from the opposite side, through the glass cover slip on which the crystals were deposited (Figure 1). The center of the diffraction limited excitation spot (which was much larger than the tip) coincided with the position of the tip.

Three different modes of the setup were used:

*Imaging Mode*: In this mode, the AFM was used in tapping mode to probe the topography of the sample with as little as possible tip–sample interaction. The cantilever was excited to oscillate and tip–sample contact only occurred at the lower turning points of each oscillation. The oscillations’ amplitude was kept constant by a control circuit that controlled the height position of the cantilever. To obtain a topographic image of a sample area, the sample was moved by the *x*‐*y* piezoelectric stage and scanned line by line. At the same time a confocal PL image of the same area was obtained.

*Local Pressure Mode (Figure* *1* *c)*: The tip was positioned above a desired location and a force–distance curve was measured. This curve (Figure 1e) describes the force applied by the cantilever, recorded as a function of the distance between the tip and the sample. (This force can be derived via the cantilever deflection and its spring constant.) The cantilever approached the sample until contact was established. Upon further movement of the cantilever toward the sample, the repulsive force was increased until a preset value was reached. In the experiments, this desired force was held constant over a time from 10 to 1000 s, while forces from 2 to 200 nN were applied. PL could be recorded simultaneously with ms time resolution from an area equal to the size of the confocal point spread function (PSF) centered around the position of the tip.

*Scratching Mode (Figure* *1* *d)*: To scratch the sample, the AFM was used in the imaging‐contact mode, however only one line was scanned. The setpoint of the cantilever deflection (respectively the force) was chosen to be high enough, to achieve irreversible sample deformation. First the tip was brought into contact with the sample, then the preset force was built up and kept constant during the 1D scanning. Forces from 40 to 500 nN were applied.

## 3 Results

**Figure 2:**
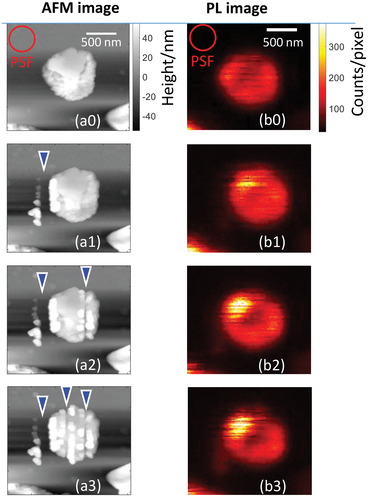
Response to cutting and scratching. Simultaneously acquired AFM and PL images of a MAPbI_3_ crystal (a0,b0) before, after the (a1,b1) first, (a2,b2) second, and (a3,b3) third scratching of its surface. The scale bar is 500 nm, the locations of scratches are marked by triangles. The circle labeled PSF shows the size of the confocal microscope point spread function. Despite scratching/cutting, the PL intensity of this microcrystal measured ≈5 min after the mechanical action was not affected (PL intensity color code is the same for all images). The sample was measured under an argon atmosphere.

Panels a0 and b0 in **Figure** **2** show AFM and PL images of a MAPbI_3_ microcrystal up to 100 nm thickness and about 1000 nm in diameter placed on a substrate and studied under an argon atmosphere. After acquiring these initial images, the crystal was scratched by the tip (see the Experimental Section). After each vertical scratch, AFM images (panels a1, a2, and a3) and PL images (panels b1, b2, ad b3) were recoded. The scratches are clearly visible from the AFM images. The first groove was so deep that the tip simply cut off a part of the crystal. The correlation between the AFM and PL images confirms that the same crystal was detected by both microscopes. Surprisingly, none of these drastic manipulations resulted in any PL intensity quenching of this crystal (PL images in (b) have the same intensity color code). An additional example is presented in Figure S2 (Supporting Information). Here, we need to note an important experimental detail: Switching from mechanical manipulation by the AFM tip to PL imaging of the crystal took at least 5 min. Thus, for the present we conclude that about 5 min after the mechanical action on the crystal, no significant effect on the PL intensity was observed.

**Figure 3:**
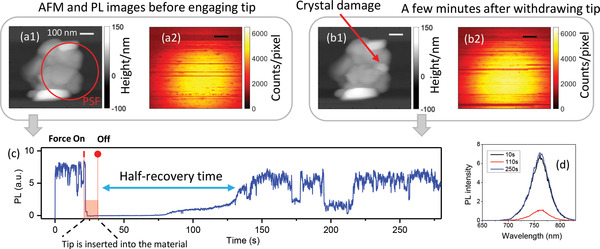
Time‐resolved response to local crystal damage by scratching. Simultaneously acquired AFM and PL images of a MAPbI_3_ microcrystal (a1,a2) before and (b1,b2) after local scratching by the AFM tip with a force of 200 nN under an argon atmosphere. The scale bar in all images is 100 nm. The red circle in (a1) is the PSF. To scratch, the sample was moved several tens of nanometers only while the tip was engaged. Note the change of the topology due to scratching shown by the arrow in (b1). (c) PL transient during the whole experiment. The PL collection area was limited by the PSF. The motion of the sample during scratching was much smaller than the PSF. The times of application and removal of the force are marked with the vertical red tick and the solid circle, respectively. (d) PL spectra (integration time 1 s) of the crystal taken at different times showing the absence of spectral shifts.

Considering that PL could potentially already recover during the delay between the mechanical action and the PL detection, we recorded PL transients simultaneously while scratching (**Figure** **3**). Before scratching of the crystal, its PL intensity shows random blinking. Within a few seconds after pushing the tip against the crystal and the initiation of scratching, the PL becomes strongly quenched. After removing the tip, the PL intensity stays at the low level for some time and then slowly recovers to the initial level after ≈120 s (the half‐recovery time, see Figure 3c). Thus, we conclude that recovery of the PL at the time scale of minutes after completing the mechanical treatment is the reason why no changes of PL were observed in the experiments shown in Figure 2. We also recorded PL spectra of the same crystal during the scratching (Figure 3d) and observed no detectable spectral changes between the initial, final, and partially recovered PL.

We note that there is one location on the crystal shown in Figure 2 where the PL becomes even stronger after scratching. One possible reason is a change in the light outcoupling conditions due to morphological changes of the surface. In addition, perovskites are known for so called PL enhancement via light soaking by deactivation of photosensitive defect states. It has been shown that PL enhancement depends on location.^[^ ^12^, ^68^ ^]^ Accordingly, the PL intensity distribution over the object may change over time. Another possibility is that some of these cut‐off nanocrystallites accidentally have a smaller number of deep defects than other nanocrystallites due to statistical fluctuations.^[^ ^36^, ^68^ ^]^

**Figure 4:**
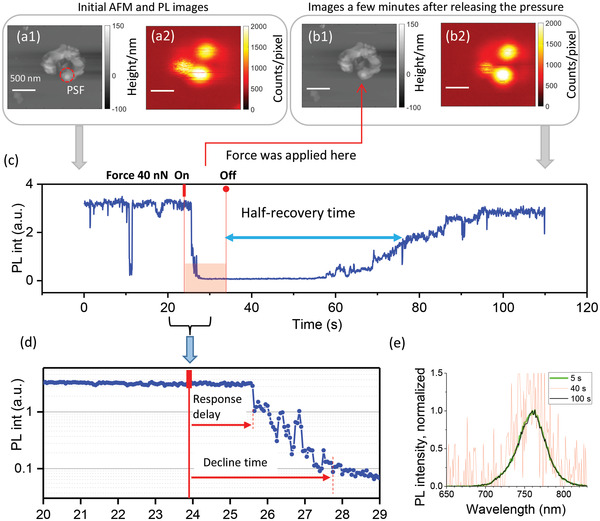
Time‐resolved response to local pressure. Simultaneously acquired AFM and PL images of a MAPbI_3_ microcrystal in air before applying (a1, a2) and several minutes after releasing the pressure (b1,b2). The scale bar in the images is 500 nm. The pressure was applied in the center of the red circle shown in (a1), the size of the circle corresponds to the PSF. (c) PL signal acquired from the area covered by the PSF. The instants of time when the force (40 nN) was applied/released are marked by the red vertical tick/the solid circle and vertical lines. (d) Zoom in of the PL drop after application of the force, note the logarithmic scale for PL intensity. The delay between application of the force and the PL response is clearly visible as well as PL fluctuations and a stepwise PL decline. (e) PL spectra of the crystal obtained at different times (integration time 1 s) showing the absence of spectral shifts. The noisy appearance of the spectrum at 40 s is due to the very weak PL.

In the next experiments we reduced the applied force to a level where it did not give any visible damage to the crystals and measured in the local pressure mode (Figure 1c). The PL of the microcrystals was monitored while forces from 2 to 200 nN were applied by the AFM tip. By using the JKR‐Model^[^ ^69^ ^]^ these forces can be translated into pressures in the order of 0.1–3 GPa (see Note S1, Supporting Information). The force was kept constant for 10 s. **Figure** **4** and Figure S3 (Supporting Information) show typical results. We found that applying local pressure qualitatively leads to the same effect as scratching of the crystal, namely: i) almost instantaneous PL quenching upon applying the force; ii) full recovery of the PL after several minutes; and iii) the whole PL dynamics is convoluted with PL blinking. It is remarkable that in the time window 20–29 shown in Figure 4d the crystal does not show any PL blinking events before the force is applied while after applying the force the PL quenching occurs via several blinking down steps.

**Figure 5:**
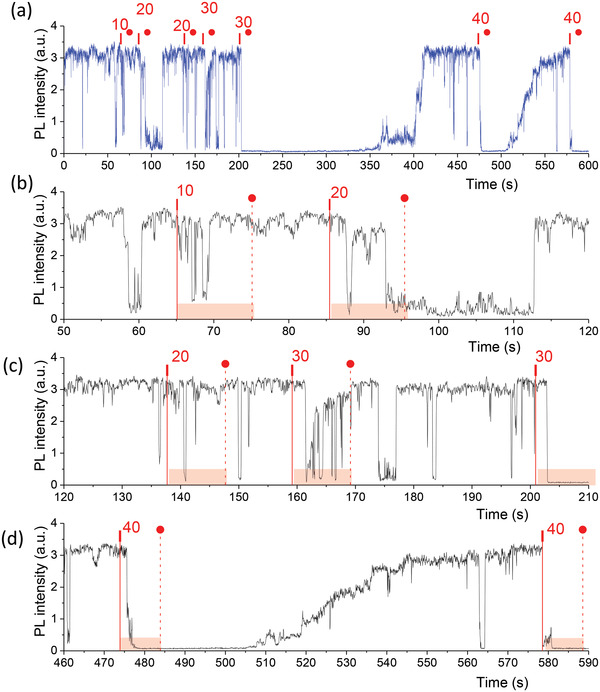
PL response of the same crystal to forces of different magnitude. PL intensity trace (in air) of the crystal shown in Figure 4. Forces of different magnitudes were applied for 10 s at different instants of time. The instants when the forces were applied/released are marked by red vertical ticks/circles and vertical lines, the magnitudes of the forces in nN are also shown. (a) Full trace, (b–d) parts of the trace with zoomed‐in time scale, (d) the same trace as in Figure 4. Application of force did not always lead to PL quenching. Note that there is always a delay between the application of the force and the PL intensity drop.

We studied the initial evolution of the PL intensity under local pressure in more detail. **Figure** **5**a shows a long‐term experiment carried out on the crystal shown in Figure 4. Several force–distance curves were recorded consecutively at an identical location of the crystal for 10 s each with different forces applied in the following sequence: 10, 20, 20, 30, 30, 40, and 40 nN. First, it can be seen that application of the same force sometimes led to quenching and sometimes it did not (see, e.g., the effects of the 20 nN force and 30 nN force). It can also be seen that both PL quenching and PL recovery are not continuous processes, rather they seem to consist of multiple steps. The initial response to the force is usually delayed and appears as jumping of the PL intensity down in the same way as in PL blinking (Figures 4d and 5). In general, one can imagine the appearance of the whole PL intensity trace being a consequence of modifications of the PL blinking pattern: application of force results in prevailing of PL “off” states over PL “on” states.

**Figure 6:**
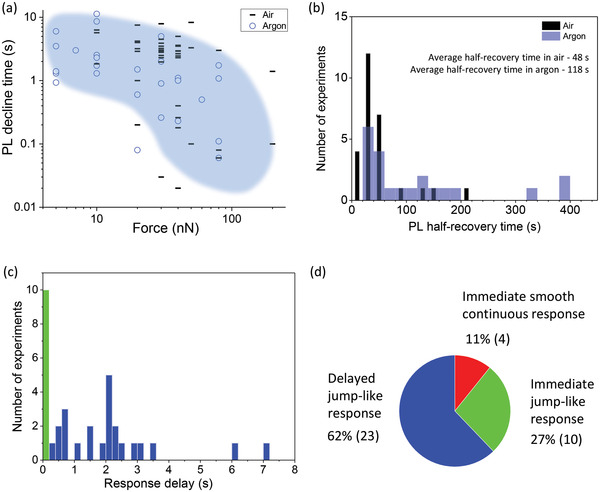
(a) Correlation between the PL decline time and the applied force. Data for all crystals (both in air and argon) are shown; the blue coloration is a guide to the eye. (b) Histogram of the PL half‐recovery times obtained in the experiments with different forces. The data for ambient air and the argon atmosphere are displayed separately. (c) Histogram of the response delay time to the applied force (for all crystals in air and argon), the green bar shows the immediate jump‐like response (delay < 5 ms). (d) Statistics of the initial response to the force, the number of cases is shown in the brackets.

To characterize the PL kinetics upon applying local pressure, we define the PL decline time as the time from the moment the force is applied until the moment when the PL drops to a stable low intensity level. We can see that a stronger force usually results in a shorter PL decline time. The overall statistics for all evaluated crystals (**Figure** **6**a) indeed shows a negative correlation between the force amplitude and the PL decline time (see also Figure S4, Supporting Information), however, some randomness of the response is also obvious.

Because the recovery lasts from tens to hundreds of seconds, which is much longer than the characteristic time of PL fluctuations,^[^ ^70^ ^]^ we are able to roughly estimate the PL half‐recovery time, defined as the time from the release of the force until the PL intensity reaches 50% of its initial value (see Figure 3c). The half‐recovery time does not have any obvious correlation with the applied force (see, for example, Figure 5a). It varies drastically from crystal to crystal and from one application of the force to another for the same crystal. Despite of these large variations and the limited number of experiments, it appears that the recovery in air is somewhat faster than in argon. The average PL half‐recovery time for MAPbI_3_ is about two times faster in ambient air (around 50 s) than in an argon atmosphere (around 100 s), see Figure 6b. This suggests that the PL recovery is promoted by the presence of oxygen or water in the atmosphere (relative humidity was around 30%).

We also analyzed the initial response of PL to the force (Figure 6c,d). In 89% of cases the PL responded to the pressure by an abrupt jump down, where in 62% of cases the jump was clearly delayed by 0.2–8 s, while in 27% of cases the response was instantaneous (faster than 5 ms, our time resolution). In the remaining 11% of cases application of the force led to an immediate but smooth continuous decline of PL without jumps.

**Figure 7:**
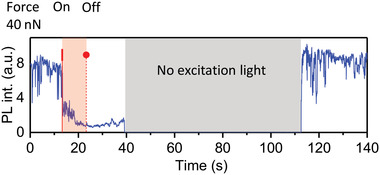
PL transient of a MAPbI_3_ microcrystal under a temporal application of a 40 nN force in argon. From 40 to 110 s the laser excitation was switched off, nevertheless, the fully recovered PL was found 70 s later by checking the PL intensity.

We also found that PL recovery does not require light illumination and occurs also in the dark. **Figure** **7** shows full recovery of the PL intensity while the crystal was kept in the dark for 1 minute. This agrees with our initial scratching experiments where several minutes after the end of scratching the initial level of the PL was always recovered despite the absence of intentional light illumination during this time.

**Figure 8:**
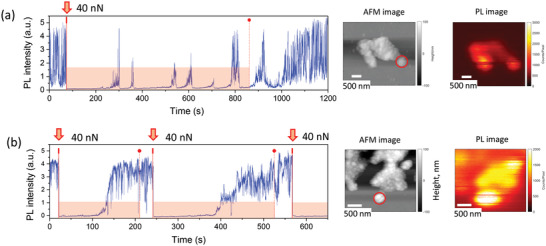
Recovery of the PL of MAPbI_3_ microcrystals under air despite a constantly applied local pressure. Time instances when force (40 nN) was applied/released are marked by red vertical tick/solid circles, vertical lines, and the reddish coloration. (a) An example of a crystal which shows several “attempts” to recover its PL under constant pressure. These “attempts” look like PL intensity flashes. (b) PL of a different crystal recovers after initial complete quenching during the force application. Three sequential applications of the force are shown. On the right side simultaneously acquired AFM and PL images of these microcrystals (before applying force) are shown. The region, where the force was applied and from which the PL was collected, is marked by red circle the size of which is equal to the PSF.

It is interesting that even under constantly applied local force in some experiments PL recovery was observed. One example of this effect can be seen in Figure 5 (force 30 nN applied at *t* = 159 s) where after the initial strong quenching the PL recovered before the force was released. To observe this effect more often, we held the force for hundreds of seconds, which is much longer than in the experiments described above where the force was held for 10 s only. **Figure** **8**a shows that upon applying the pressure, the PL first was completely quenched, however, after about 150 s the PL intensity started to increase despite of the force being still held constant. When the PL reached its initial value at *t* = 300 s it suddenly dropped again. The whole process looks as “attempts” of the crystal to recover its PL which, however, were only partially successful. Several more of such “attempts” can be seen in the curve until the pressure was finally released at *t* = 850 s allowing complete PL recovery. Cases of full PL recovery under pressure were also observed as illustrated in Figure 8b. Simultaneously acquired AFM and PL images of the samples are shown on the right side in Figure 8. PL recovery under pressure appears as a series of light flashes with higher and higher amplitude until something triggers the complete PL quenching again.

## 4 Discussion

### 4.1 Nonlocal Response to a Local Force: Role of Charge Diffusion

We observed that the response of the PL to the localized mechanical force spread over a much larger volume of the crystal than one would expect considering the size and the anticipated interaction region of the AFM tip. Indeed, while only the top surface of the sample was mechanically addressed, the PL excited and collected from the opposite side (glass) of the sample was affected. This fact alone can be explained by considering that 640 nm excitation light has a quite large penetration depth (1/e attenuation over 200 nm for MAPbI_3_), which is often larger than the measured thickness of the studied crystals. The lateral dimension of the region where the PL was quenched was at least of the size of the PSF (400 nm in diameter) which again is much larger than the diameter of the tip/sample contact area (20–75 nm^2^, Note S1, Supporting Information). This illustrates that local stress can perturb the charge dynamics of the whole crystal of several hundreds of nanometers in size. For example, the PL of the 500 nm sized crystal shown in Figure 3 was completely quenched by local damage visible in the AFM image as a small bright spot (see Figure 3b, 1). Indeed, the strain induced by the tip spreads over a larger area than the tip/sample contact region, thereby potentially affecting the charge carrier dynamics. After all, probably the main effect responsible for the widespread impact of the small tip sample interaction region is the large charge carrier diffusion length in MAPbI_3_. Upon photoexcitation charges can travel over at least several hundreds of nanometers during the PL lifetime^[^ ^71^ ^]^ making the mechanically affected area accessible for charge carriers photogenerated in a large volume around this area. Remarkably, the same mechanism seems to be behind PL blinking in large semiconductor nano‐ and microcrystals.^[^ ^24^ ^]^

### 4.2 Phenomenological Model Based on Viscoelasticity and the PL Dependence on Strain

If we do not consider PL blinking for a moment, the overall dependence of the PL on force looks like the dependence of strain (deformation) of a viscoelastic solid on an applied force. In a viscoelastic solid it takes time to build up strain under constant stress and it also takes time for the strain to disappear after the stress is removed. Literature data show that MHPs possess viscoelastic properties.^[^ ^73^ ^]^ Accordingly, it takes time for the PL to become quenched (for the solid to deform) under the force, and it takes even longer time for the PL to rise again (for the deformation to disappear) after the force is removed. The Voigt model^[^ ^74^ ^]^ of a viscoelastic solid allows to calculate the time evolution of strain *ε* under a time‐dependent stress *σ* (force)

(1) $$\eta \frac{d}{\mathrm{dt}}\varepsilon \left(t\right)+k\varepsilon \left(t\right)=\sigma \left(t\right)$$

where *η* and *k* are the viscosity and elasticity coefficients, respectively. In this model application of a constant force leads to an exponential increase of strain. When the force is removed, the strain decreases exponentially. Both processes are described by one characteristic time *τ* = *η*/*k*.

**Figure 9:**
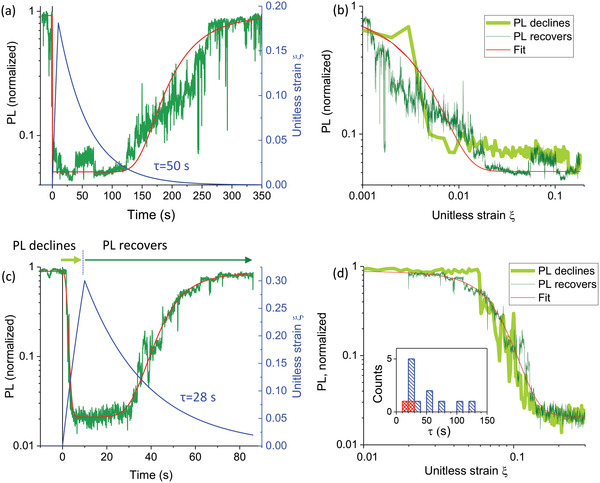
Theoretical modeling of the PL response to applied local pressure for two crystals. (a,b) Fitting of the data for one crystal, c,d) for another crystal. Panels on the left (a, c) show the experimental PL intensity (dark green), a fit of the PL intensity (red) and the unitless strain (blue) as functions of time. Unitless strain is $\xi =\frac{k}{\sigma_{0}}\varepsilon$, see Note S3, Supporting Information, for details. The strain increases from *t* = 0 (pressure is applied) until *t* = 10 s (pressure is released), after that the strain relaxes to zero. The panels to the right (b, d) show the PL dependence on the unitless strain during the initial quenching (PL declines, light green line) and during the recovery (PL recovers, dark green line). Ideally these two dependencies should be identical. The calculated PL‐dependencies are also shown by red lines. Fitting parameters for the crystal shown in (a,b)‐characteristic time of strain relaxation *τ* = 50 s, *A* = 9.6, PL_0_ = 0.051, *ξ*_0_ = 0.0025, *β* = 10; and for the crystal shown in (c,d): *τ* = 28 s, *A* = 0.9, PL_0_ = 0.021, *ξ*_0_ = 0.018, *β* = 0.03. The inset to panel (d) shows the distribution of the strain relaxation times *τ* obtained from fitting of 12 PL response curves among which 10 were measured in argon (blue) and 2 in air (red).

We propose that the PL intensity unambiguously depends on the crystal strain *ε*.^[^ ^51^ ^]^ This dependence PL(*ε*) must be the same when the crystal is under pressure and when the crystal relaxes after the force was removed, because it passes through the same range of *ε*: from zero to *ε*_max_ during the PL decline and from *ε*_max_ to zero during the PL recovery. Thus, by matching the PL decline and PL recovery trajectories in the coordinates of *ε*, we can find the characteristic time *τ* of the Voigt model. If the two trajectories match each other, it means that the theory can describe the experimental data. Indeed, the experimentally measured trajectories (PL decline and recovery) satisfy this condition quite well. By analyzing the shape of the obtained experimentally PL(*ε*) dependencies for different crystals, we found that it can be approximated by the following formula

(2) $$\begin{array}{ccc} \mathrm{PL} & = & \mathrm{PL}\left(\varepsilon \right)+{\mathrm{PL}}_{0}=A\frac{k_{r}}{k_{r}+k_{\mathrm{nr}}\left(\varepsilon \right)}+{\mathrm{PL}}_{0}\\ & = & \frac{A}{1+\beta \exp(\varepsilon \left(t\right)/\varepsilon_{0})}+{\mathrm{PL}}_{0} \end{array}$$

where PL_0_ is the PL coming from the part of the crystal, which is not affected by the quenching; and *k*_r_ and *k*_nr_(*ε*) are effective radiative and nonradiative rates, respectively, in the part of the crystal affected by the force. *β* gives the strength of the force‐induced NR recombination in comparison with the radiative recombination. *ε*_0_ is the strain, the application of which increases NR recombination by *e* times, see the details in Note S3 (Supporting Information). We fitted 12 PL trajectories with this model, two of them shown in **Figure** **9**. We found that the characteristic strain relaxation time is distributed from 10 to 120 s with most of the data belonging to the 20–60 s interval, see inset in Figure 9d. Figure 6b shows a similar range for the half‐recovery time. The range of the characteristic times extracted from our experiments matches well the results from ref. [73] where the strain relaxation in perovskite single crystals was measured mechanically and the authors applied a more complex Voigt model with two characteristic times around 5 and 50 s, respectively. The parameters *β* and *ε*_0_, however, vary from crystal to crystal much more broadly. This is not surprising considering the stochastic nature of the PL intensity of such small objects in general (see Section 4.4). If we assume that the PL response is determined by just a few metastable NR centers, whose status is dependent on the strain, the whole response should be dependent on the exact location of the tip relative to the NR centers. This makes the parameters *β* and *ε*_0_ randomized. Interestingly, the obtained relaxation times in the range of 10–100 s match the typical timescale of transient processes like PL bleaching and PL enhancement in MHPs.^[^ ^12^, ^70^, ^75^ ^]^

In the framework of this theory, the PL decline time (see Note S3, Supporting Information) is inversely proportional to the applied force, which generally agrees with the correlation observed experimentally (Figure 6a). As for the PL recovery time, it depends on force only logarithmically (see Note S3, Supporting Information). As this dependence is weak, it is in line with the absence of any clear correlation between the force and the PL half‐recovery time.

### 4.3 Possible Mechanisms behind the Strain Dependence of the PL

Next, let us discuss possible microscopic mechanisms behind the phenomenological dependence of the PL on strain [Equation (2)]. The PL intensity of a semiconductor depends on many parameters like charge trapping rates, radiative recombination rates, rates of various Auger process, and so on. For MAPbI_3_ this dependence can be described by the modified Shockley–Read–Hall (SRH+) model.^[^ ^60^ ^]^ When the PL goes up or down under constant light illumination, some parameters of the SRH+ model change during the macroscopic observation time *t* of the experiment.

The presence of PL blinking (abrupt changes of the PL intensity) also indicates that some parameters of the SHR+ model (or any other recombination model) are stochastic and change abruptly during the macroscopic observation time *t*. The currently accepted picture of PL blinking in MHP microcrystals assumes that each crystal contains a small number of metastable NR centers possessing very large charge capturing and recombination cross sections (Figure S1, Supporting Information).^[^ ^24^ ^]^ Following the literature,^[^ ^26^ ^]^ we call them supertraps to distinguish from other defects. Activation of just one supertrap leads to an immediate and substantial drop of the PL intensity. Consequently, the NR rate introduced by just one supertrap can be even higher than the NR rate due to all other defects which are present in the crystal before the supertrap becomes active. More details on this issue are presented at the end of Section 4.

Without loss of generality, we can assume that there are two types of defects: common defects (number of these defects in one microcrystal *N* ≫ 1) with rather low charge recombination cross section and very few supertraps in the active state (their number *N*_s_ < 10) with very high capturing and recombination cross sections. *N*_s_ is small and it fluctuates over time causing PL blinking.^[^ ^24^, ^26^, ^36^, ^76^ ^]^ So, all parameters except *N*_s_ are continuously changing parameters, while *N*_s_ is a small integer number and thus changes only in relatively large steps (e.g., from 0 to 1, from 1 to 2, and so on).^[^ ^36^ ^]^

Let us split all possible scenarios of the origin of the PL quenching under stress to three cases:

Case 1: Dynamics of *N*_s_ is not affected, but at least one parameter in the SRH+ is affected by strain.

Case 2: Dynamics of *N*_s_ is affected by strain; all other parameters are constant.

Case 3: Dynamics of *N*_s_ is affected by strain and at least one other parameter is affected too.

There is an important experimental fact allowing to exclude Case 1. It is the presence of a time delay between the instant of application of force and the instant change of the PL intensity. This delay ranges from 0.2 to several seconds and it was observed in 62% of the data (see Figures 4 and 5 and statistics in Figure 6c,d). Because the strain starts to develop as soon as the force is applied (see Figure 9), there should be no delay of the material response. Thus, if strain changes any of the continuous parameters of the model, an immediate change of the PL should be visible. The expected response is an immediate drop of the PL, or an immediate start of its continuous decline, all of which contradict the majority of experimental observations.

Case 2, however, does not contradict the delayed response of the material. Because switching of the supertraps from active to inactive (passive) states is random and rather rare, it does not need to happen exactly at the time when the force is applied. Let us consider a simple model for illustration (see an example in Note S4, Supporting Information). Imagine that strain stabilizes the active state of the supertraps (increases the switching time from active to inactive state) but does not affect much the switching time from the inactive to active state. In this case, the response of the crystal to strain cannot be faster than the switching time of the supertraps. Thus, it takes time until one inactive super‐trap jumps to its active state. Upon continuous application of force, the strain‐induced stabilization of the supertraps’ active state causes an increase of the number of active supertraps in the crystal and, thus, a decrease of the PL. When the force is removed, the strain slowly relaxes and the equilibrium between the active and inactive states shifts accordingly. Thus, the number of supertraps in active state decreases leading to PL recovery. To elaborate on this possibility, we consider in Note S4 (Supporting Information) a specific model of the supertrap switching and show that it can indeed lead to the observed behavior of PL under strain.

Case 3 is unlikely, because an immediate response of the PL should be still observed as in Case 1. Case 3 is only possible if the effect of the modification of the continuous parameters on the PL intensity is quite small in comparison with the effect of adding one active supertrap, which basically reduces it to Case 2.

From the above we can conclude that application of force very likely changes the switching dynamics of the supertraps leading to the presence of a larger number of these species in their active state in the strained crystal. So, Case 2 appears as a very likely explanation of the phenomenon at least in 60% of studied crystals.

In the theory based on viscoelasticity we assumed that the PL depends on strain. In this case the strain build‐up and relaxation times determine how fast the system reacts on the application and removal of the force. This model can be combined with the idea described in Case 2 that strain stabilizes the active state of the supertraps. For the time‐averaged PL dynamics to be determined solely by strain, as it is assumed in this theory, the switching of the supertraps must be faster than the strain relaxation time. This agrees with the experiments: The maximum characteristic time of blinking is around 1 s,^[^ ^70^ ^]^ while the strain relaxation times are in the range of 20–140 s (Figure 9).

One can also look at the strain‐based theory of the PL response from a different point of view: Build‐up and relaxation of strain is a consequence of rearrangements of the lattice. These processes inevitably result in defects dynamics, including their formation, annihilation, motion, and transformation. The experimentally observed time scales would be compatible with slow defect relaxation due to, e.g., ion diffusion. Supertraps, as all other defects, take part in this overall evolution, which results in supertrap switching dynamics to be dependent on strain. In this picture the metastable defects (supertraps) should be affected in the first place, because they possess active and inactive states to switch between, while it is more difficult to change nominally stable defects.

After discussing several scenarios to interpret our observations, for the sake of completeness we finally want to exclude another seemingly obvious possibility. In principle, slow recovery after removal of force could be solely explained by the slowness of the supertrap switching. In this case, we would not need to involve strain as a slow‐changing parameter at all. We simply could assume that the equilibrium between the active and inactive states of the supertraps depends on force (stress) instead of strain. Contrary to strain, force can be applied and removed instantaneously. When the force is removed, the system becomes instantaneously nonequilibrated. To reach the equilibrium takes time, because the switching time from the active to inactive state is slow. However, such a scenario is unlikely, because as we discussed above, the maximum characteristic time of blinking in MAPbI_3_ is of the order of 1 s, which is far too short to account for 100 s recovery time often observed experimentally.

### 4.4 Recovery Attempts under Applied Force

The discussion presented above cannot easily account for the recovery attempts and full recovery under stress (and strain) observed in a few cases (Figures 5 and 8). Obviously, under specific circumstances the material can accommodate the effect of the strain on charge recombination and return to its initial PL intensity state even when the force is still applied. This is a very drastic illustration of the self‐healing ability of MAPbI_3_. We speculate that at the location where the force is applied, the material locally rebuilds in such a way that the region around the tip simply does not participate in electron‐hole dynamics any longer. For example, the crystal structure could change locally to a different phase with a higher bandgap compared to tetragonal MAPbI_3_ at room temperature. Indeed, it has been shown that under a relatively low pressure of 0.3 GPa there is a phase transition toward the higher bandgap orthorhombic phase.^[^ ^41^ ^]^ In this case the strain induced NR centers activated close to the tip may become isolated in this new high bandgap phase and their contribution to the NR processes in the whole crystal will decrease. Presumably, the charge carries will quickly escape this high bandgap region to the surrounding (cubic or tetragonal phase) where the bandgap is lower. The same mechanism has already been proposed to explain the highly inhomogeneous spatial distribution of PL intensity and PL enhancement in MAPbI_3_ microrods during the tetragonal–orthorhombic phase transition region at around 160 K without pressure.^[^ ^77^ ^]^ Note that the force of 40 nN applied in the experiments shown in Figure 8 translates to a pressure of around 0.3 GPa (Note S1, Supporting Information). Therefore, a connection of the PL recovery under applied force to the phase transition indeed seems possible. Yet, these are still tentative ideas, and we hope that future experiments will deliver additional insights.

### 4.5 Beyond Ensemble Averaging, a Large Diversity of Responses Is Expected

Our experiments demonstrate that different microcrystals show very individual responses of their PL on the applied force (Figures 5 –8). At a first glance it may seem like poorly reproducible results and even question the proposed physical picture of the underlying processes. However, to the contrary, broadly distributed responses are expected. The reason of this is fundamental. It is because we carry out the experiments beyond ensemble averaging with small individual objects as samples. The response of each crystal is determined by random transitions of a small number of individual systems (small number of stochastic variables, for example, the states of several supertraps), where the transitions not only depend on the local environments on the nanoscale, but also occur at highly nonequilibrated conditions (so‐called nanoscale thermodynamics).^[^ ^78^ ^]^ Theoretically, averaging of all these responses over tens of thousands of objects and force application attempts (thus over all possible configuration coordinates like local configurations of supertraps, their distances to the tip, the morphologies of the crystal surface at the location of the tip, sizes of the crystals, peculiarities of local charge carrier diffusion, and many others) should lead to a response as one would measure in an ensemble. However, it is difficult to imagine an experiment which would directly give the ensemble‐averaged result of local force applications to small crystals. By studying small crystals individually, we deliberately avoid ensemble averaging to directly detect activation/deactivation of a single NR center. This gives us a much deeper insight on the processes and allows discussing mechanistic explanations of the otherwise ensemble‐averaged phenomena like PL intensity increase/decrease and self‐healing. So, we stress that the differences between the behaviors of different crystals do not contradict to the proposed explanation of the effect, on the contrary, they support it.

### 4.6 Dependence on the Atmosphere

It is well documented that light‐induced transient effects in MAPbI_3_ depend on the surrounding atmosphere.^[^ ^12^, ^68^, ^79^ ^]^ The effect of a particular atmosphere can be either PL enhancement or PL decline depending on the stoichiometry of the sample.^[^ ^80^ ^]^ Different atmospheres also lead to very different PL blinking behaviors of MAPbI_3_ crystals.^[^ ^81^ ^]^ Accordingly, it is not surprising that we found statistically different PL recovery times of MAPbI_3_ crystals in air and in argon atmospheres. The role of oxygen and water in perovskite degradation and defect evolution has been discussed in numerous publications. Several plausible chemical reactions, involving the ions composing the perovskite and water and oxygen from the environment, have been proposed^[^ ^82^ ^]^ However, despite of these efforts it is still not possible to pinpoint with confidence the nonradiative recombination centers which are induced/inhibited by these reactions.^[^ ^12^, ^83^, ^84^, ^85^ ^]^ Our experiments indicate that the presence of air accelerates the return of the system to the initial equilibrium, which might be related to an enhanced ion diffusion in the presence of oxygen and water (or H^+^) in the crystal. Note that since we observed PL recovery without light illumination, the diffusion of ions that leads to rebuilding of the crystal structure is not necessarily light driven.^[^ ^12^, ^68^, ^83^, ^86^ ^]^

### 4.7 Nature of the Supertraps

Although there is compelling evidence supporting the presence of NR centers of different types with very different charge capturing and recombination cross sections, and their type and concentration depend on sample preparation, environment, light irradiation, and many other factors, it is not yet possible to pinpoint their exact chemical nature. Electrical experiments also give quite different picture of the defect types and concentrations in comparison with pure optical measurements.^[^ ^87^, ^88^ ^]^ Numerous theoretical calculations suggest that iodide interstitials and atomic lead (Pb^0^) are deep defect states, which are expected to provide high charge trapping and recombination rates.^[^ ^88^, ^89^ ^]^ In addition, defect complexes (in analogy to donor–acceptor pairs) whose association and dissociation could provide switching have been suggested. The simplest complex is a Frenkel defect pair—an iodide interstitial and an iodide vacancy.^[^ ^90^ ^]^ However, there is a problem in direct assigning the strong NR recombination centers to iodide interstitials or Frenkel pairs. Many XPS experiments have shown that in the few nm thick region near the surface of MAPbI_3_ the I/Pb ratio is around 3.6 instead of 3.0 expected from a perfect stoichiometry.^[^ ^80^ ^]^ This implies that thousands of iodide interstitials in the near‐surface region of a 100 nm × 100 nm × 100 nm crystal should be expected, while for PL blinking only a few metastable NR centers per crystal are needed. Based on this reasoning it can be concluded that the numerous iodide interstitials located close to the surface cannot be the quenchers we are looking for. There have been several distinct experiments suggesting that defect clustering is common in perovskites. Along these lines supertraps are not just single defects, but rather clusters thereof, which are responsible for NR recombination and material degradation.^[^ ^91^, ^92^, ^93^ ^]^ Defect clusters may have very different properties than the single defects they are composed of. Accordingly, it is not obvious that a cluster of deep defects promotes any NR recombination. At the same time, it is also not apparent that a cluster of benign defects (when isolated) stays benign. For example, a donor–acceptor pair (with both defects being quite shallow and not inducing high recombination rates when isolated) can be a strong nonradiative recombination center because it can efficiently capture both charge carriers at a very small spatial location.^[^ ^26^ ^]^ Discussing these various options, we have to admit that the chemical nature of the supertraps still remains an open question.

### 4.8 Importance of the Metastable NR Centers for Perovskites

The phenomenological model of metastable supertraps in MHPs readily explains many transient effects of MHPs.^[^ ^24^, ^25^, ^26^, ^27^, ^28^, ^29^, ^30^, ^31^, ^35^ ^]^ Although we cannot yet tell their chemical nature, we know with confidence that the supertraps are extremely efficient NR centers in comparison to any others. Indeed, for the PL intensity to drop by a factor of two by adding one supertrap, the induced NR rate should be at least equal to the total recombination rate (the radiative rate plus NR recombination rate due to all other NR centers already present before adding of the supertrap).^[^ ^26^, ^36^ ^]^ Considering this, we would like to explicitly formulate the following general question: Are there any other effective NR centers in MAPbI_3_ semiconductor except of the metastable supertraps?

We have the following crucial observations: i) PL blinking in MAPbI_3_ individual crystals^[^ ^24^, ^26^ ^]^ and films^[^ ^34^, ^35^, ^68^ ^]^ and ii) PL quenching under strain likely occur due to stabilization of supertraps in their active state. These two observations strongly suggest that the metastable NR centers are indeed responsible for the majority of NR recombination in MAPbI_3_. This conclusion agrees well with the remarkable matching between the typical trap concentration obtained from PL studies of MAPbI_3_ thin films^[^ ^18^, ^60^, ^88^, ^94^ ^]^ and the concentration of the metastable NR centers in blinking crystals prepared by the same deposition methods (10^15^–10^16^ cm^3^, corresponding to several NR centers per nano/microcrystal).^[^ ^26^, ^36^, ^95^ ^]^ Thus we can state that at the moment there are no experimental facts contradicting the hypothesis that a substantial if not the major part of strong NR recombination centers in MAPbI_3_ are metastable, while there are indications in favor of this hypothesis. Therefore, we finally reaching a match between the peculiar defect tolerance and self‐healing properties observed in bulk samples and devices and the fluctuation processes observed at the level of individual nano‐ and microcrystals. Remarkably, the self‐healing of the PL as well as its degradation and recovery can be explained by the evolution of supertraps and other metastable defects which switch from one state to the other in response to a change of the environmental conditions. Moreover, metastability of NR centers is a factor leading to apparent defect tolerance, which was not considered before. Indeed, despite of the existence of NR centers, their time‐averaged contribution to the overall NR recombination can be much less than one would expect form their concentration and trapping cross sections simply because the centers are active only a fraction of time due to their switching.

## 5 Conclusion

Correlative atomic force and confocal fluorescence microscopy allowed us for the first time to directly probe the influence of mechanical stress and local damage on charge recombination processes in MAPbI_3_ perovskite. Despite of the initial severe PL quenching triggered by the mechanical force applied by the AFM tip, complete self‐recovery of the initial PL properties of polycrystalline MAPbI_3_ microcrystals was always observed at a time scale of minutes. Indeed, while strain and crystal damage promote very efficient nonradiative charge recombination, this state of the material is not permanent. The semiconductor always returns to the initial balance between radiative and nonradiative charge recombination. The self‐healing process does not require light irradiation and depends on the surrounding atmosphere. The PL intensity of the studied crystals showed pronounced blinking. Upon application of the force, the PL intensity in >60% of cases dropped after some delay in just one or a few discrete abrupt steps in a similar manner as it fluctuates during PL blinking. These observations suggest that strain stabilizes the active (strongly quenching) state of the metastable NR recombination centers compared to the inactive state. Therefore, the whole PL quenching and recovery process is mostly driven by the dynamics of metastable NR charge recombination centers, the presence of which is corroborated by the PL blinking caused by them. The data suggest that it is highly likely that majority of efficient NR recombination centers in MAPbI_3_ semiconductor are metastable contributing to the defect tolerance property of the material. We propose a phenomenological model where the NR rate depends on strain induced by stress according to the Voigt model of viscoelasticity and found the characteristic time of strain relaxation in MAPbI_3_ to be on the order of 10–100 s. The ability of MAPbI_3_ to recover after mechanical damage is an extraordinary property for a crystalline semiconductor. It can be highly advantageous for future demanding applications of these materials.

## Conflict of Interest

The authors declare no conflict of interest.

## Author Contributions

J.L. prepared the samples, participated in the experiments, did the initial data analysis, and took part in the coordination of data collection, and preparing of the first draft of the paper. M.G. carried out all experiments, collected all data, and participated in writing of the manuscript. IS designed the concept of the study and wrote most of the paper. P.F. developed the theory, modeled the experimental data, and wrote the theoretical part of the paper. T.B. participated in writing the manuscript and, together with IS, designed the experiments and coordinated the project. P.F. and I.S. with support of T.B. developed the physical interpretation of the experimental data.

## Supporting information

Supporting information

## Data Availability

The data that support the findings of this study are available from the corresponding author upon reasonable request.
