# Supplementary material for: Self‐Healing Ability of Perovskites Observed via Photoluminescence Response on Nanoscale Local Forces and Mechanical Damage
Source: Adv Sci (Weinh). 2022 Dec 1;10(1):2204393. doi: 10.1002/advs.202204393 (PMC9811431; doi:10.1002/advs.202204393)
Supplement: Supplementary file 1 — Supporting information [file ADVS-10-2204393-s001.pdf]

ADVANCED SCIENCE
Open Access

Supporting Information

for *Adv. Sci.*, DOI 10.1002/advs.202204393

Self-Healing Ability of Perovskites Observed via Photoluminescence Response on Nanoscale Local Forces and Mechanical Damage

*Marco H. J. J. Galle, Jun Li, Pavel A. Frantsuzov, Thomas Basché and Ivan G. Scheblykin**

Supplementary Information

Self-healing ability of perovskites observed via photoluminescence response on nanoscale local forces and mechanical damage.

Jun Li[1†], Marco H. J. J. Galle[2†], Pavel A. Frantsuzov[3], Thomas Basché[2], and Ivan G. Scheblykin[1,*)]

[1]Chemical Physics and NanoLund, Lund University, Box 124, 22100, Lund, Sweden
[2] Institute of Physical Chemistry, Johannes Gutenberg-University, Duesbergweg 10-14, 55128, Mainz, Germany
[3]Voevodsky Institute of Chemical Kinetics and Combustion, Siberian Brunch of the Russian Academy of Science, Institutskaya 3, Novosibirsk 630090, Russia

*) *ivan.scheblykin@chemphys.lu.se*
† These authors contributed equally to this work.

Table of content

Supplementary Figure 1. Supertrap model of PL blinking.

Supplementary Figure 2. Additional example of PL recovery after local scratching.

Supplementary Figure 3. Additional example of PL response to local pressure.

Supplementary Figure 4. Response of PL intensity to the local force of different magnitudes applied to the same crystal.

Supplementary Note 1. Estimation of the pressure from the force applied by the tip and the contact area.

Supplementary Note 2. Theoretical model based on visco-elasticity.

Supplementary Note 3. PL decline time and half-recovery time in the framework of the theoretical model based on visco-elasticity

Supplementary Note 4. A possible model for the switching of the NR centres which can explain the experimental data.

Supplementary Note 5. Sample identification numbers for the record.

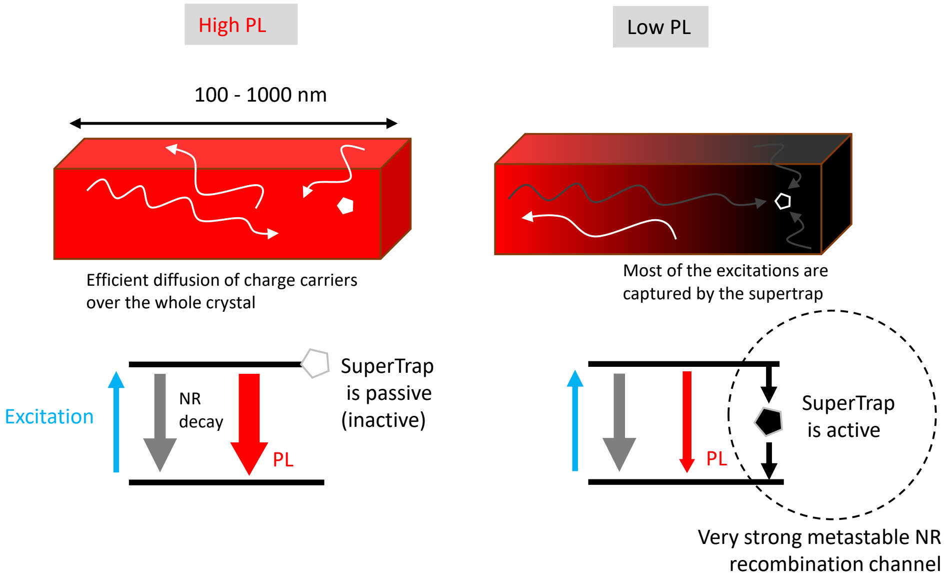

Supplementary Figure 1. Supertrap model of PL blinking.

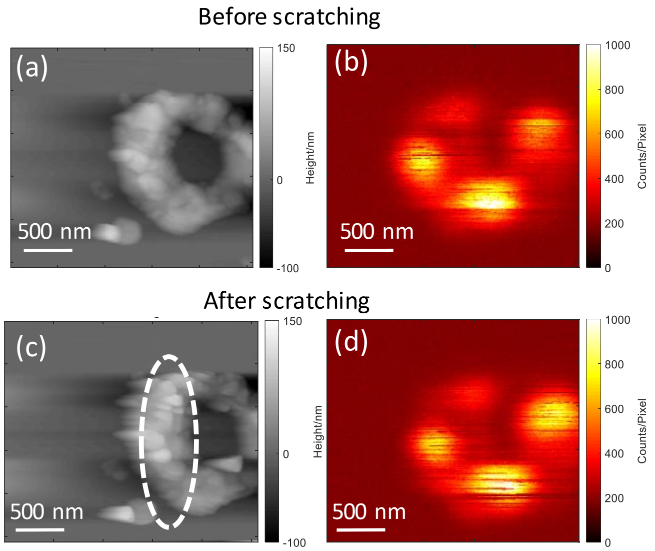

Supplementary Figure 2. Additional example of PL recovery after local scratching. AFM and PL images of a $MAPbI_3$ polycrystal before (a, b) and after (c, d) scratching in an argon atmosphere. The scratched region is marked by a dashed white oval.

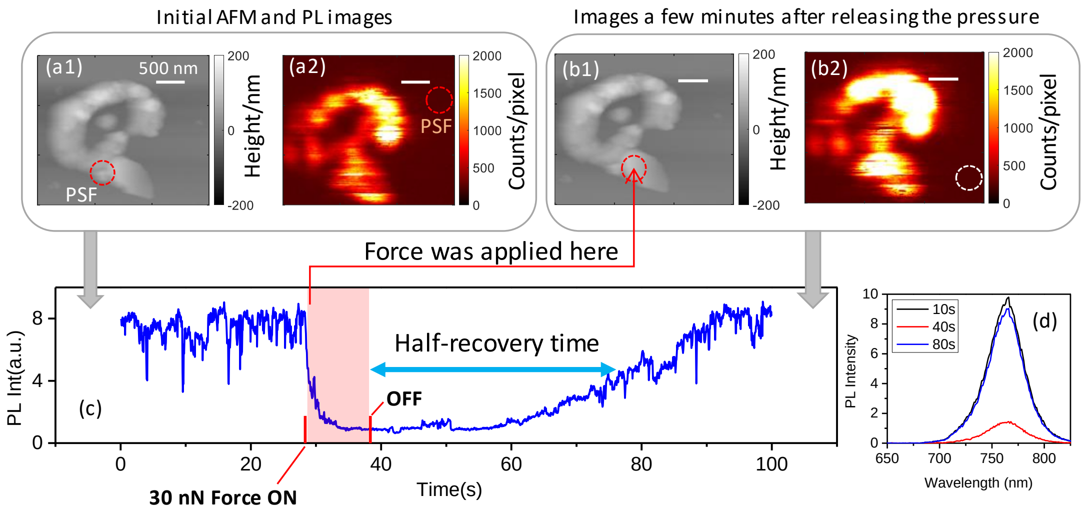

Supplementary Figure 3. Additional example of PL response to local pressure. AFM and PL images of a $MAPbI_3$ microcrystal before (a1,a2) and several minutes after releasing the pressure. The scale bar is 500 nm. The point of pressure application is marked by an arrow in (b1). (c) The PL signal was measured in the confocal regime at the point of force application. The signal was integrated over the area covered by the PSF, the diameter of which is shown by the red circle (430 nm). The instants of time when the force (30 nN) was applied and released are marked by the vertical red ticks. (d) PL spectra of the crystal obtained at different times showing the absence of spectral shifts. Sample ID: 20191014006, in argon.

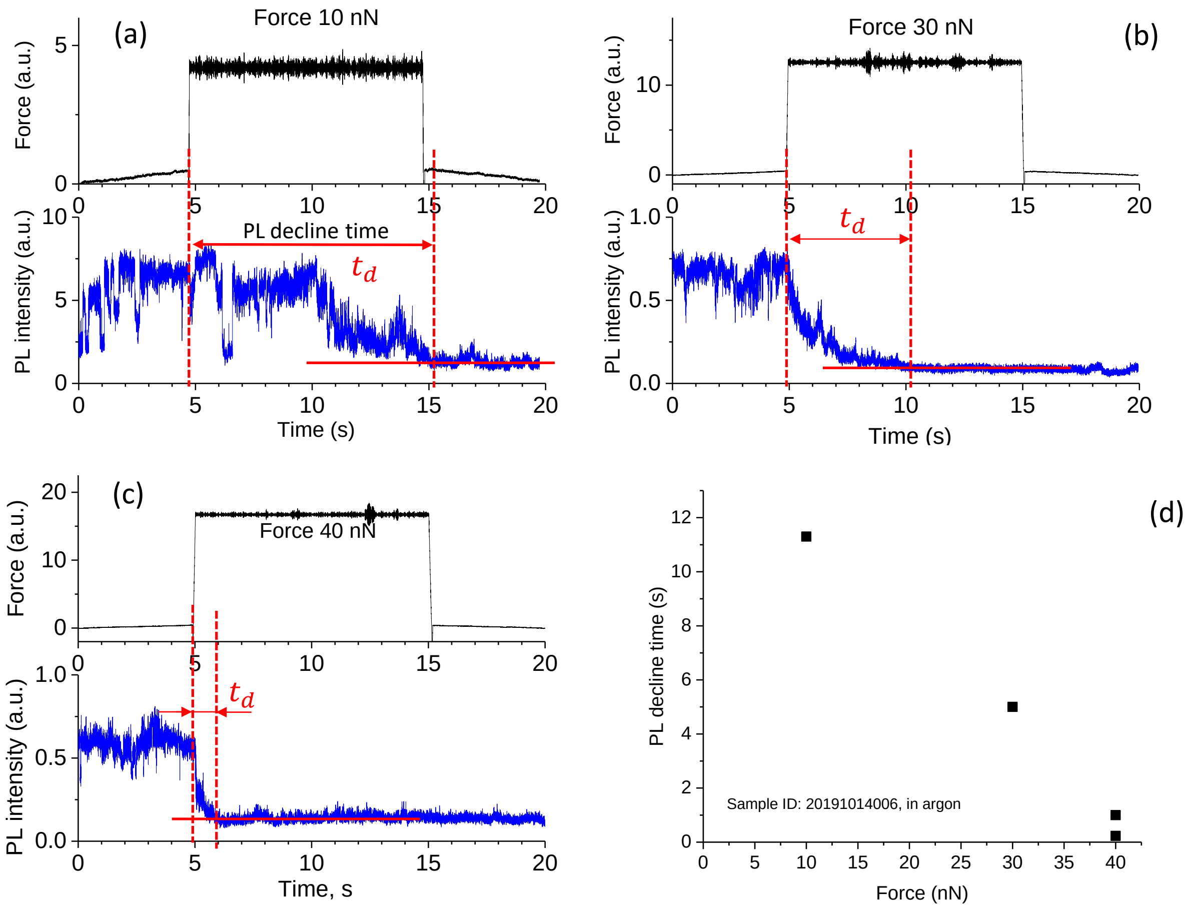

Supplementary Figure 4. Response of the PL intensity to local forces of different magnitude applied by the AFM tip to the same crystal. (a), (b) and (c) show the PL as a function of time (bottom) in comparison to the applied force (20, 30 and 40 nN) as a function of time (top) for the crystal shown in Supplementary Figure 3. Red lines define the PL decline time t_d for each PL trace. Panel (d) shows the dependence of the PL quenching time on the applied force (defined in (a)) obtained for this crystal. Sample ID:20191014006, in argon.

Supplementary Note 1. Estimation of pressure from the force applied to the tip and the contact area.

To estimate the effective pressure from the applied force, we have employed the Johnson-Kendall-Roberts (JKR) model,[1] which is considered to be applicable for soft samples with large adhesion forces between tips and samples and large tip sizes.[2] Since no large tips were used, our treatment is only a rough estimate. The reduced Young's modulus was calculated using the elastic modulus E = 157.5 GPa and a Poisson's ratio ν = 0.27 for silicon,[2] as well as E = 13.9 GPa and ν = 0.33 for $MaPbI_3$.[3] As tip radius, 7 nm (given by the manufacturer) was used. The adhesion force was roughly estimated from the experimentally observed, average pull-off-forces of the force-distance-curves to be ~10 nN. Based on these assumptions, contact radii of 2.4-4.9 nm and contact areas of

20-75 nm^2, respectively, were derived. Thus, a force regime of 2 – 200 nN corresponds to pressures of 0.1-3 GPa.

Supplementary Note 2. Theoretical model based on visco-elasticity

The basic assumption of the model is that PL intensity directly depends on crystal strain ε. The time dependence of applied stress σ in the experiment when force is applied at time 0 and released at time t_{off} is given by

$$\sigma(t) = 0\,,\ \text{for}\ \ t < 0$$
$$\sigma(t) = \sigma_0\,,\ \text{for}\ \ 0 < t < t_{\text{off}}$$
$$\sigma(t) = 0\,,\ \text{for}\qquad t_{\text{off}} < t \qquad \text{(S4)}$$

Assuming that the perovskite nanocrystal can be described by the Voigt model[4] one can write the equation for the strain time dependence $\varepsilon(t)$

$$\eta \frac{d}{dt}\varepsilon(t) + k\varepsilon(t) = \sigma(t) \qquad \text{(S5)}$$

where k is elasticity coefficient and η is the viscosity. Solving Eq.S5 with the stress dependence Eq.S4 we have:

$$\varepsilon(t) = \frac{\sigma_0}{k}\left[1 - \exp\left(-\frac{t}{\tau}\right)\right]\,,\ \text{for}\ \ 0 < t < t_{\text{off}}$$
$$\varepsilon(t) = \frac{\sigma_0}{k}\left[1 - \exp\left(-\frac{t_{\text{off}}}{\tau}\right)\right]\exp\left(-\frac{t - t_{\text{off}}}{\tau}\right)\ ,\ \text{for}\ \ t_{\text{off}} < t \qquad \text{(S6)}$$

where τ is relaxation time of strain:

$$\tau = \frac{\eta}{k} \qquad \text{(S7)}$$

We assume that the PL intensity directly depends on the strain:

$$PL(t) = F(\varepsilon(t)) \qquad \text{(S8)}$$

where the function $F(\varepsilon)$ must be found from the experimental data.

Let's introduce the unitless strain $\xi = \frac{k}{\sigma_0}\varepsilon$

$$\xi(t) = \left[1 - \exp\left(-\frac{t}{\tau}\right)\right]\,,\ \text{for}\ \ 0 < t < t_{\text{off}}$$

$$\xi(t) = \left[1 - \exp\left(-\frac{t_{\text{off}}}{\tau}\right)\right]\exp\left(-\frac{t - t_{\text{off}}}{\tau}\right)\ ,\ \text{for}\ \ t_{\text{off}} < t \qquad \text{(S9)}$$

Let us fit the experimental PL intensity as a function of theoretical $\xi(t)$ given by (S9) for both PL decline (when the force is applied) and PL recovery when the force is removed with the same fitting parameter τ. It turned out that indeed our experimental data can be fitted in this way (see Figure 9 in the main text).

The obtained $PL(\xi)$ dependence can be approximated by the following function:

$$PL(\xi) = \frac{A}{1+\beta\exp(\xi/\xi_0)} + PL_0 \qquad \text{(S10)}$$

Thus, the time dependence $PL(t)$ can be now approximated by the formula

$$PL(t) = \frac{A}{1+\beta\exp(\xi(t)/\xi_0)} + PL_0 \qquad \text{(S11)}$$

were $\xi(t)$ is given by Eq.S9. The results of the fitting for two different crystals are shown in the main text in Figure 9.

Supplementary Note 3. PL decline time and half-recovery time in the framework of the theoretical model based on visco-elasticity

PL decline time t_d is determined as the time passed from the moment of force application until the PL intensity reaches a stable low intensity level. It means that within this time the first term in Eq.S11 becomes much smaller (for example 20 times less) than its initial value at $t = 0$. We can write this condition as:

$$\frac{A}{1+\beta\exp(\varepsilon(t_d)/\varepsilon_0)} = \frac{1}{20}\frac{A}{1+\beta}$$

or

$$\varepsilon(t_d) = \varepsilon_0 \ln\left(20 + \frac{19}{\beta}\right)$$

The dependence $\varepsilon(t)$ given by Eq.S6 can be approximated for small t by a linear function:

$$\varepsilon(t) = \frac{\sigma_0}{k\tau} t$$

From the last two equations we obtain:

$$t_d = \frac{\varepsilon_0}{\sigma_0} k\tau \ln\left(20 + \frac{19}{\beta}\right) \qquad \text{(S12)}$$

So, the PL decline time is inversely proportional to the applied force.

Half-recovery time t_r is defined as the time interval from t_{off} until the moment when PL recovers to 50% of its initial value. Mathematically we can write this condition as:

$$\frac{A}{1+\beta\exp(\varepsilon(t_{\text{off}}+t_r)/\varepsilon_0)}=\frac{1}{2}\frac{A}{1+\beta}$$

or

$$\varepsilon(t_{\text{off}}+t_r)=\varepsilon_0\ln\left(2+\frac{1}{\beta}\right)$$

By substituting this to Eq.S6 we obtain the expression for the half-recovery time:

$$t_r=\tau\ln\left[\frac{\varepsilon_0 k}{\sigma_0}\left[1-\exp\left(-\frac{t_{\text{off}}}{\tau}\right)\right]^{-1}\ln\left(2+\frac{1}{\beta}\right)\right] \qquad \text{(S13)}$$

So, in the model based on viscoelasticity t_r is very weakly (logarithmically) dependent on the applied force which agrees with the experimental observations.

Supplementary Note 4. A possible model for the switching of the NR centres, which can explain the experimental data.

In this model we realize the idea presented in the paper that the switching dynamics of the metastable NR centres is affected by the strain and this effect only causes reversible PL quenching.

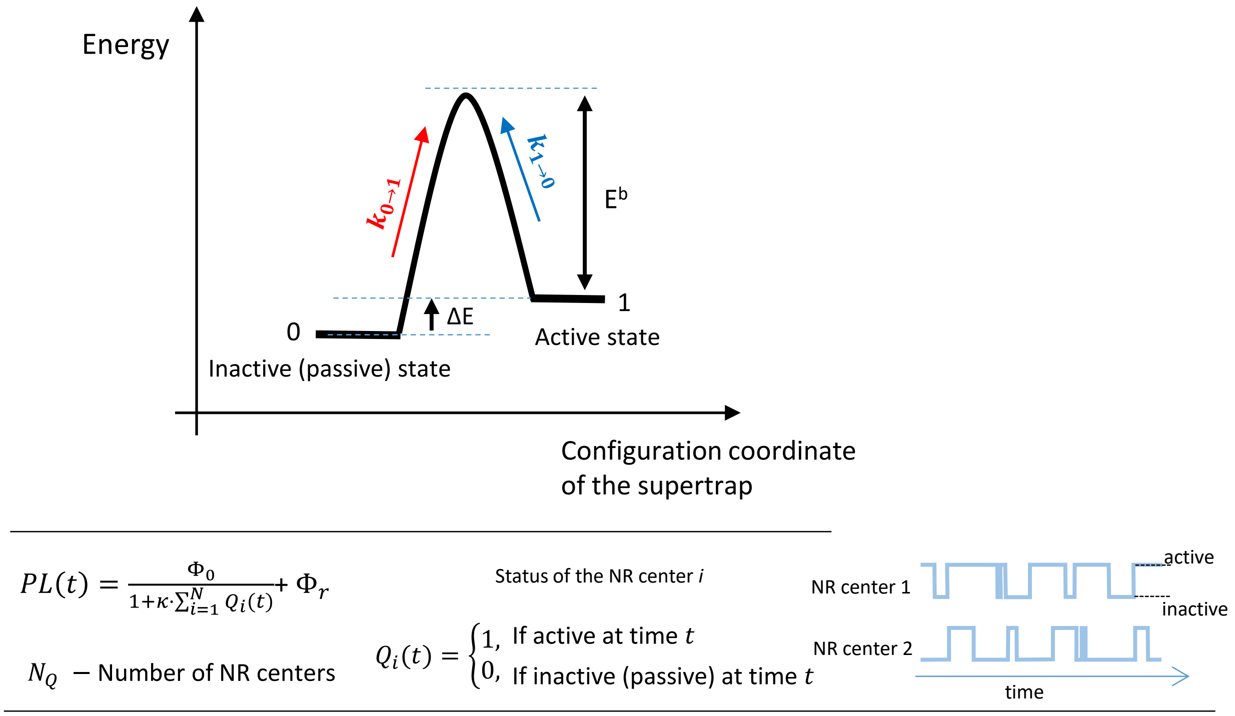

Supplementary Figure 5. Configuration space of a metastable non-radiative center. One of many possible models. PL is quenched by the center when it is in active state.

In the presence of N non-radiative centres, the PL intensity of a semiconductor crystal is determined by stochastic dynamics of the variables $Q_i(t)$, $i=1\ldots N$, each of them can abruptly

change its value from 0 (inactive state of the centre, no NR recombination occurs) to 1 (active state of the centre, NR recombination rate is equal to κ). Let us assume the simplest dependence of the PL intensity on the activity of the centres (Supplementary Figure 5):

$$PL(t) = \frac{\Phi_0}{1+\kappa\sum_{i=1}^{N} Q_i(t)} + \Phi_r \tag{S14}$$

where Φ_r is the emission intensity from the rest of the crystal not being affected by stress, $\Phi_0 + \Phi_r$ is the emission intensity when all metastable centers are inactive and κ is ratio between the nonradiative rate of the recombination by one NR center and the radiative recombination rate.

Let us assume that for each NR centre the rates of switching from 0 to 1 ($\gamma_{0\to1}^i$) and from 1 to 0 ($\gamma_{1\to0}^i$) are determined by thermal activation:

$$\gamma_{0\to1}^i = \gamma_0 \exp\left(-\frac{E_i^b + \Delta E_i}{kT}\right)$$

$$\gamma_{1\to0}^i = \gamma_0 \exp\left(-\frac{E_i^b}{kT}\right)$$

Where E_i^b – the hight of the barrier $1 \to 0$ for the centre *i* and ΔE_i is the energy difference between the configuration states 1 and 0, see Supplementary Figure 5. A broad distribution of E_i^b gives the multi-timescale character of the PL blinking dynamics. Averaged value of Q_i over the switching dynamics is:

$$\langle Q_i \rangle = \frac{1}{1 + \exp(\Delta E_i/kT)}$$

Let us assume that the energy difference between state 1 and state 0 for each NR centre depends linearly on strain (and thus on time in our experiments with temporal applying of local force) and this dependence is the same for all centres:

$$\Delta E_i(t) = \Delta E_0 - kT\frac{\varepsilon(t)}{\varepsilon_0}$$

Where ΔE_0 - energy difference for center *i* at zero strain. The dependence $\varepsilon(t)$ is given by eq. (S6). Substituting in Eq. (S14) each $Q_i(t)$ by $\langle Q_i \rangle$, we obtain:

$$\langle PL(t) \rangle \approx \frac{\Phi_0}{1 + \dfrac{\kappa N}{1 + \exp\left(\frac{\Delta E_0}{kT}\right)\exp(-\varepsilon(t)/\varepsilon_0)}} + \Phi_r$$

This is not an exact equality, it is an approximation, because the used procedure is not a formally exact averaging. After some re-arrangement we obtain:

$$\langle PL(t) \rangle \approx \frac{\Phi_0}{\kappa N + 1}\ \frac{\kappa N}{1 + (\kappa N + 1)\exp\left(-\frac{\Delta E_0}{kT}\right)\exp(\varepsilon(t)/\varepsilon_0)} + \frac{\Phi_0}{\kappa N + 1} + \Phi_r$$

By introducing designations

$$PL_0 = \frac{\Phi_0}{\kappa N + 1} + \Phi_r$$

$$A = \frac{\Phi_0 \kappa N}{\kappa N + 1}$$

$$\beta = (\kappa N + 1)\exp\left(-\Delta E_0 / kT\right)$$

we find a dependence which is analogues to Eq. (S11):

$$\langle PL(t) \rangle \approx \frac{A}{1+\beta \exp(\varepsilon(t)/\varepsilon_0)} + PL_0 \quad \text{(S15)}$$

So, in the framework of this particular model, when we average over the fast blinking dynamics, we obtain the same dependence of the PL over time Eq. (S15) as in the pure phenomenological theory based on visco-elasticity (Eq.(S11)).

Supplementary Note 5. Sample identification numbers:

Figure 3. Sample ID: 20191126014 in argon.

Figure 4. Sample ID: 20191023003 in air.

Figure 5. Sample ID: 20191023003, in air.

Figure 7. Sample ID: 20191014006, 40 nN, in argon.

Figure 8. (a – Sample ID: 20191118005 in air, b – 20191119002, in air).

Figure 10. (a) and (b) – Sample ID S2019102200201 in argon,

(c) and (d) - Sample ID S2019102300303 in argon.

Supplementary Figure 3. Sample ID: 20191014006, in argon.

Supplementary Figure 4. Sample ID:20191014006, in argon.

Supplementary references

[1] S. P. Section, B. Railways, T. Centre, S. Physics, *Proc. R. Soc. London. A. Math. Phys. Sci.* **1971**, *324*, 301.

[2] H.-J. Butt, B. Cappella, M. Kappl, *Surf. Sci. Rep.* **2005**, *59*, 1.

[3] Y. Rakita, S. R. Cohen, N. K. Kedem, G. Hodes, D. Cahen, *MRS Commun.* **2015**, *5*, 623.

[4] J. C. Jaeger, *Elasticity, Fracture and Flow with Engineering and Geological Applications*, Springer Netherlands, Dordrecht, **1971**.
